# Transition from pediatric to adult nephropathic cystinosis care: the structure, challenges and lessons learned

**DOI:** 10.3389/fped.2025.1584257

**Published:** 2025-06-12

**Authors:** Brianna Borsheim, Andrew Vissing, Cybele Ghossein

**Affiliations:** ^1^ Division of Pediatric Nephrology, Department of Pediatrics, Northwestern University Feinberg School of Medicine, Ann & Robert H. Lurie Children's Hospital, Chicago IL, United States; ^2^ Division of Nephrology and Hypertension, Northwestern University Feinberg School of Medicine, Chicago, IL, United States

**Keywords:** cystinosis, nephropathic cystinosis, transition of care, pediatric nephrology, health care transition program

## Abstract

Cystinosis is a rare, autosomal recessive disorder that results in a build up of the amino acid cystine in the body ( 1). With early diagnosis and advances in patient prognosis over the years, this has led to an increasing number of adolescents and adults with cystinosis. Multiple studies have shown that adolescents and young adults (YA) with kidney disease transitioning to adult care are at high risk for poor health outcomes ( 1– 4). In addition, patients with cystinosis have cognitive and psychosocial struggles that may interfere with their health care autonomy. Pediatric and adult nephrologists often act as the care-quarterback for patients with cystinosis at the time of transition. Northwestern Medicine (NM) Nephrology has implemented a formal program for the transition of care for young adults with kidney disease from Lurie Children's Hospital to Northwestern Medicine. This multidisciplinary team has assisted in the transition of several patients with nephropathic cystinosis since its inception. There are a myriad of challenges that arise as patients with cystinosis transition from pediatric to adult care including inadequate resourcing, loss of continuity and lack of adult expertise in rare pediatric diseases. While there is no universally accepted definition of transition success, the process should ensure uninterrupted care, address evolving medical needs and support patients’ autonomy and self-advocacy in adulthood.

## Introduction

Cystinosis is a rare, autosomal recessive disorder that results in a buildup of the amino acid cystine in the body (1). Previously, cystinosis was a fatal pediatric condition characterized by poor growth, Fanconi syndrome, and kidney failure by 10-years of age (2). However, with the introduction of cystine-depleting therapy and advancements in kidney transplantation, cystinosis has evolved from a fatal pediatric condition to a chronic multi-organ disorder. This advancement in care has led to an increasing number of adolescents and adults with cystinosis, which has accelerated the need for guidelines related to the health care transition (HCT) from pediatrics to adult care.

Kidney disease continues to be the hallmark of cystinosis with extrarenal manifestations becoming more apparent over time. Most young adult (YA) patients with cystinosis have chronic kidney disease (CKD) or are kidney transplant recipients. Given the universality of kidney disease, nephrologists are an essential part of the HCT from pediatric to adult care. With the improvement in treatment and life expectancy, cystinosis represents a quintessential rare pediatric renal condition that requires a model for guided transition to adult care.

### Cystinosis in adolescence and young adulthood

Cystinosis is a multiorgan disease with two main subtypes—infantile nephropathic cystinosis and juvenile nephropathic cystinosis (1, 3). Infantile nephropathic cystinosis is the most common subtype, representing ∼95% of all cases, and presents in early childhood with severe Fanconi syndrome with progression to CKD stage 5 by 10–12 years of age. The juvenile nephropathic form has slower progression and impacts a smaller group of patients (∼5%) (1LE). It presents with proteinuria and a mild tubulopathy, but can still progress to CKD stage 5 in adulthood (1).

**Figure 1:**
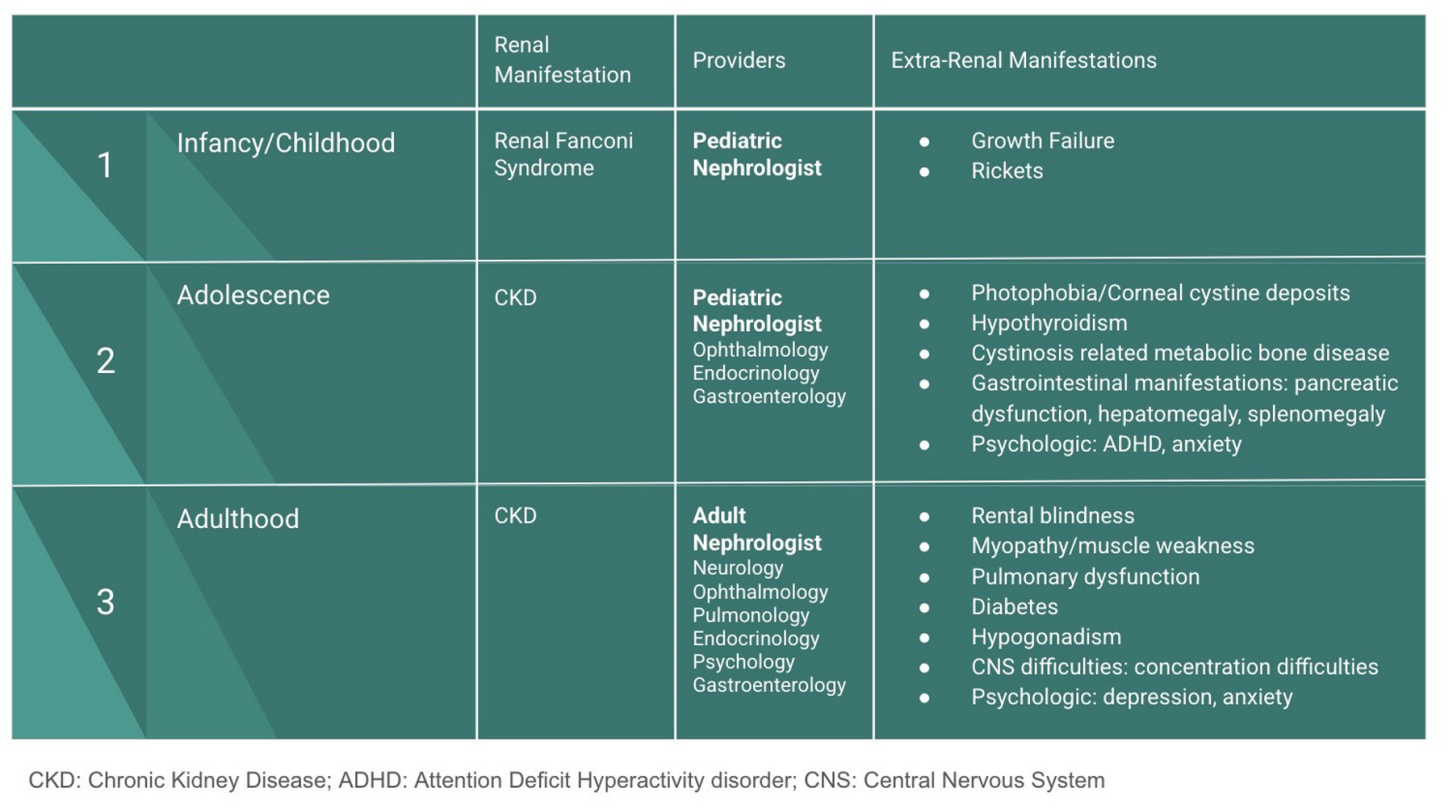
Symptoms of nephropathic cystinosis and approach to multidisciplinary care.

Through treatment with cystine-depleting therapy and advancements in kidney transplantation, cystinosis has become a chronic, progressive condition impacting patients throughout adolescence and adulthood. In addition to renal involvement, cystinosis can impact various other organ systems. Ocular manifestations are characterized by corneal cystine deposition. Gastrointestinal symptoms and endocrine complications, such as hypothyroidism, are common within the first decade of life (1). Adolescents and YAs often experience bone disease, termed cystinosis-related metabolic bone disease, which can lead to bone deformities, pathologic fractures or osteoporosis, and pain (4). Among other manifestations detailed in Figure 1, young adult patients often develop neurologic symptoms which include neurocognitive impairment and academic difficulties (4) (Figure 1).

Furthermore, as young patients with cystinosis approach the HCT to adult care, it is crucial to acknowledge the significant psychologic and social difficulties associated with cystinosis. Health-related quality of life (HrQoL) is lower in pediatric patients with cystinosis compared to their healthy peers (5, 6). Studies have found patients with cystinosis to have low adherence to medical care, emotional distress, anxiety, depression, school difficulties, and attention deficit disorder (7). Recognition of the systemic nature of cystinosis and understanding the physical and psychosocial aspects of the disease is critical for a successful transition from pediatric to adult care.

## Transition to adult care

The pediatric-to-adult transition for patients with chronic disease is often poorly managed and disjointed (8). This transition is especially challenging for adolescents and young adults with rare, systemic conditions. Young adulthood is a period of critical development, but it is also a period of fragility. Patients are asked to take on responsibilities for their health during a time of transition and disruption in their educational, vocational, and psychosocial life (9).

Multiple studies have shown that adolescents and YA with kidney disease are a high-risk group for poor health outcomes due to a myriad of reasons (10–13). Furthermore, YA with cystinosis have cognitive and psychosocial struggles which may interfere with the development of their health care autonomy (4, 5). Studies have shown that patients, families and physicians identify the transition period as a critical time in cystinosis disease progression (14). The influx of responsibilities in conjunction with the psychosocial burdens can lead cystinosis patients to become non-adherent, leading to the development of comorbidities, transplant graft loss, as well as education, social, and mental health struggles (14, 15). Understanding and caring for the complex needs of YA with cystinosis requires a formal, multidisciplinary approach.

Below, we present a young man's transfer to adult nephrology via a specialized transition clinic aimed at overcoming some of the known HCT challenges. Northwestern Medicine Nephrology has implemented a formal program for the transition of care for YA with kidney disease from Anne & Robert H Lurie Children's Hospital (LCH) to Northwestern Medicine (NM). This transition clinic is operated by adult nephrologists but located within the pediatric care center. While this model is not entirely novel, it remains uncommon, with few documented examples of transition programs structured this way. This clinic has assisted with the transition of several patients with nephropathic cystinosis since its inception in 2016 (16). This clinic primarily serves patients with chronic kidney disease or patients post-kidney transplant with the vast majority being transplant patients. Patients with nephropathic cystinosis on dialysis transition through the dialysis unit.

Cystinosis is a rare genetic disorder, and historically, affected individuals did not survive into adulthood. As a result, adult nephrologists had limited exposure to managing these patients. However, the advent and widespread use of cystine-depleting therapy has significantly improved life expectancy, thereby creating a new adult patient population requiring ongoing nephrology care. To prepare for this evolving need, our adult nephrology team gained experience in cystinosis management through close collaboration with pediatric nephrology, participation in specialized cystinosis seminars, and by establishing a national network of care partners with expertise in this condition.

### The structure—a patient’s transition

#### Diagnosis and management in pediatric care

The patient is a 21-year-old male who was diagnosed with nephropathic cystinosis at 14-months of age. He initially presented with vomiting, constipation and failure to thrive at 12 months of age. The diagnosis of nephropathic cystinosis was made via mixed leukocyte cystine level testing (2.08 nmol ½ cystine/mg protein; normal <0.2 nm ½ cystine/mg protein) and genetic analysis. He was started on oral immediate-release (IR) cysteamine bitartrate (CYSTAGON®; Viatris Inc, Canonsburg, PA, USA) after diagnosis. He was found to have cystine crystal corneal deposits and started on cysteamine eye drops when they became commercially available.

During childhood, the patient developed Fanconi syndrome, requiring long-term fluid and electrolyte supplementation. He continued with slow growth and weight gain, requiring nutritional assistance via a gastric-tube (G-tube) from 14 months to 5 years of age. His medical history is also notable for stage 3a CKD with proteinuria. Additionally, he has had complications from his cystinosis including significant muscle weakness, metabolic bone disease resulting in deformities, growth failure requiring growth hormones, delayed puberty and hypothyroidism. At the pediatric center, he was followed by endocrinology, ophthalmology, and neurology for manifestations of his cystinosis.

#### Transition

**Figure 2:**
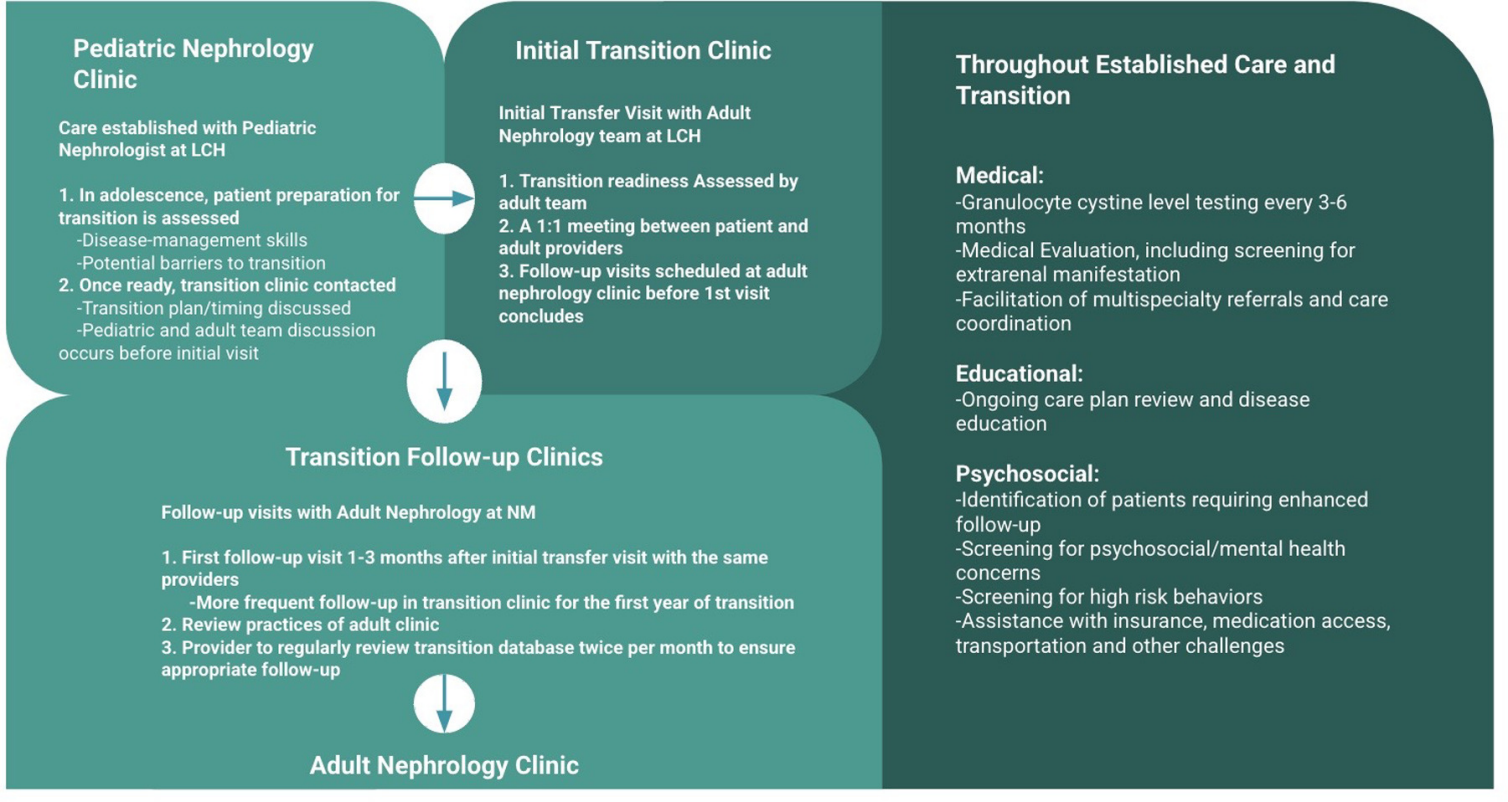
Our care model at lurie children's hospital (LCH)/northwestern medicine (NM) for transitioning patients with cystinosis from pediatric to adult nephrology (16, 24).

At 21 years of age, this patient was referred by his pediatric nephrology team to our adult transition clinic. Prior to this, he received approximately 1 year of transition preparation, which included assessments of his disease understanding, medication knowledge, self-management skills. Transition Readiness Assessment Questionnaire (TRAQ) was used to aid in this patient, his caregiver and the provider team in the transition process. The overall transition process was discussed with the patient and a multidisciplinary team and appropriate transition time was determined. Before his initial transition appointment, a care conference between the pediatric and adult nephrology teams was completed and the patient's medical history and potential barriers to transition were discussed (Figure 2).

At his first transition visit, the patient met with the adult nephrologist, physician assistant, and social worker at the pediatric site (LCH). He had the opportunity to meet each of the providers with his mother as well as one-on-one. He continued to fill out the TRAQ to assess progression and the adult nephrology clinic protocols were shared. The adult care team reviewed differences between pediatric and adult care, which included potential changes to medication and laboratory schedule. During this visit, he was scheduled for his first follow-up visit at the adult facility (NM) within 3 months. He attended all subsequent visits at the adult facility, and his follow-up attendance was monitored.

Additionally, this patient was involved with national organizations focused on cystinosis. He continued to be an involved member and drew support from this group through the transition process. This patient has now completed his transition to the adult nephrology clinic and continues to be seen every 6 months. His laboratory parameters, including kidney function, remain stable and he continues to take his medication as prescribed. He has not yet required kidney transplantation.

**Figure 3:**
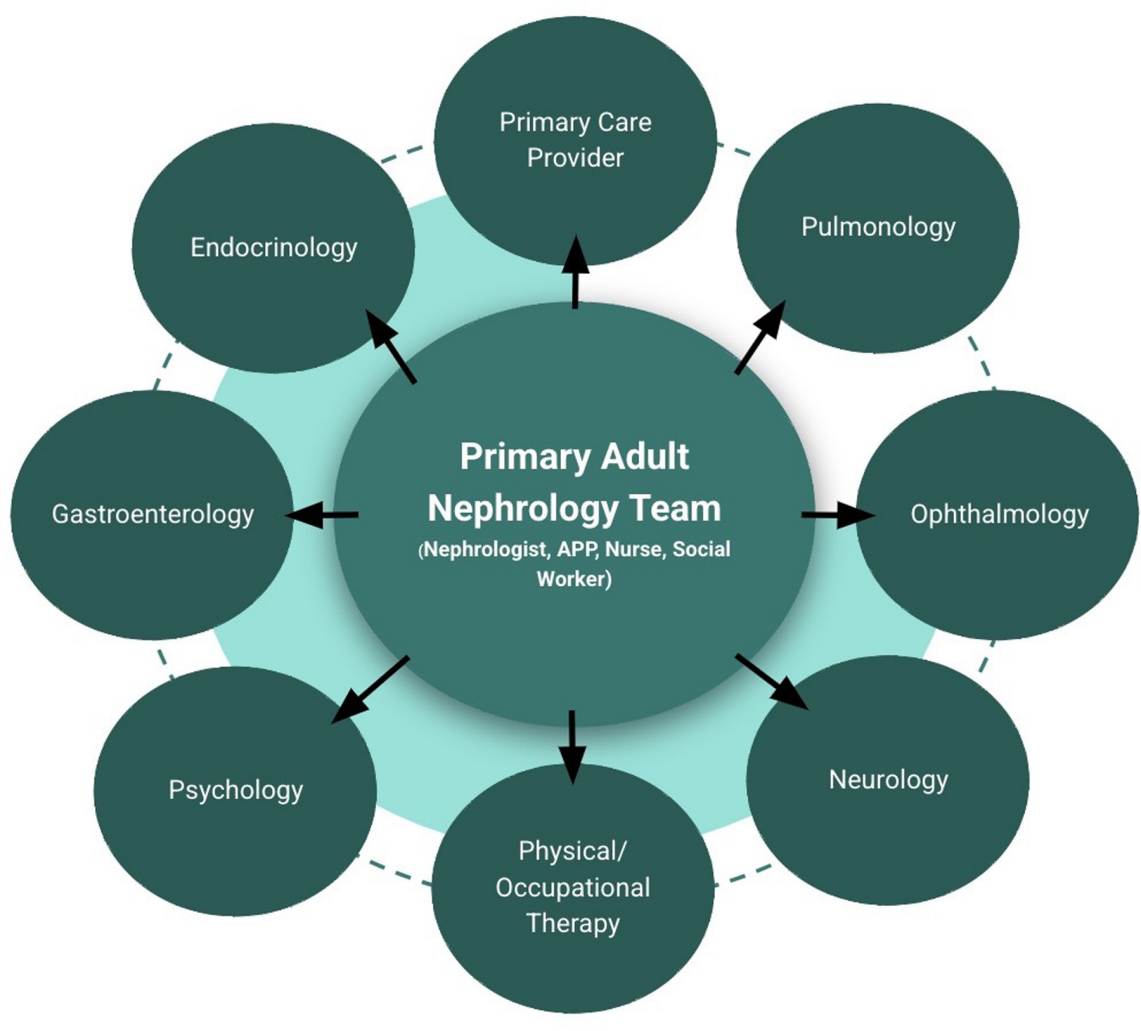
A schematic of our model with the primary adult nephrology team as the care quarterback for patients with cystinosis.

The nephrology team at NM acts as this patient's “care quarterback” after transition—facilitating as-needed referrals to an adult primary care provider and adult specialists while also coordinating multidisciplinary communication for this patient (Figure 3). Upon transition, this patient's primary nephrologist connected with the patient's new primary care provider and placed referrals to ophthalmology, endocrinology, gastroenterology and physical therapy. His nephrology team monitored him for additional endocrine, pulmonary, and neuromuscular complications and anticipated the need for referrals to the appropriate specialists as needed.

## The challenges

There are a myriad of challenges that arise as patients with nephropathic cystinosis transition from pediatric to adult care. In general, rates of transition preparedness are low. The 2018 National Survey of Children's Health revealed that only 16.2% of all youth with childhood-onset conditions (age 12–17) received the necessary services for transition to adult care (17). One of the first challenges faced is how to define “preparedness” for transition. White & Cooley (2018) defined preparedness as the extent to which a clinician: (1) speaks with the patient alone during preventive visits; (2) works with the youth to develop self-care skills and understand health care responsibilities; and (3) discusses the transition to an adult provider with the patient (18). Transition timing and skill development should be individualized and coordinated. As illustrated in the patient scenario above, allowing sufficient time for transition preparation is critical to a successful transition to adult care.

In addition to these general considerations, nephropathic cystinosis presents a set of unique challenges due to its complex, multisystemic nature. While kidney disease dominates early management, cystinosis also impacts the eyes, thyroid, pancreas, gastrointestinal system, muscles, and brain. The progression and timing of these complications requires life-long, coordinated surveillance. Adult nephrologists–especially those without training in rare pediatric-onset conditions–may be unfamiliar with the natural history of cystinosis or its extrarenal manifestations, which can lead to fragmented care and missed opportunities for early intervention (3, 19).

Another major issue is the risk of non-adherence to cysteamine therapy, which is a cornerstone of disease management. As these patients assume more responsibility for self-management during the transition, adherence often declines, especially in the absence of continued support from caregivers and pediatric providers. This can have devastating effects on disease progression and quality of life.

Furthermore, psychosocial and identity-related challenges are central during this period. Adolescents and YA with cystinosis must navigate the tension between seeking independence and managing a lifelong dependency on healthcare. This may lead to emotional distress, anxiety about disease progression and depression. Additionally, patients may struggle to adapt to the adult healthcare environment, which is typically less integrated and supportive than pediatric care. The absence of a multidisciplinary model and reduced availability of transition-trained providers can further alienate patients during a vulnerable time.

Moreover, pediatric and adult care is delivered differently. Pediatric clinics typically offer more coordinated, family-centered services whereas adult clinics may be more resource-constrained, more disease-specific, and focused on individual rather than holistic care. A 2024 study by Vissing et al. found that for both pediatric and adult nephrologists, limited resource availability was the main perceived obstacle to establishing a transition clinic (20). This study also found that adult providers often lacked interest or expertise in managing YA patients with complex pediatric-onset conditions such as cystinosis (20).

As outcomes improve and patients live longer, adult providers will increasingly encounter individuals with cystinosis. Concentrating patients with specific providers or clinics and identifying a “transition champion” can help build familiarity and expertise (20). As these patients transition care, the adult nephrologist becomes the key player, coordinator of care, and often the first point of contact for these patients. Structured handoffs and interprofessional communication are essential to maintaining continuity and reducing the risk of disengagement.

Lastly, though there is no universally accepted definition of a successful transition, most frameworks agree that the process should support patient autonomy, self-advocacy, and uninterrupted, high-quality care (9, 16). The consequences of a poor transition are significant and well described for patients with chronic kidney disease (10, 12, 14, 21, 22). Poor transitions are associated with treatment non-adherence, loss to follow-up, and both physical and psychological decline (4, 14). In contrast, successful transitions have been shown to reduce renal function decline and lower rates of acute rejection in transplant recipients (22). Despite these benefits, barriers to establishing robust transition programs persist, including logistic, operational, and financial challenges. Few studies have examined the long-term cost-effectiveness of transition infrastructure, representing an important area for future research.

## Lessons learned

Through caring for patients with cystinosis in our Lurie Children's Hospital/Northwestern Medicine nephrology transition clinic, we have identified multiple key strategies that contribute to a successful transition from pediatric to adult care. Our experience, combined with emerging evidence has helped shape a more comprehensive understanding of what effective transition entails.

### Early integration between pediatric and adult providers builds trust

A core component of our model is early collaboration between pediatric and adult nephrologists. During the transition period, adult providers are introduced to patients within the pediatric setting, allowing both parties to build rapport in a familiar environment. This overlapping care period reduces anxiety and strengthens continuity. A 2016 coordinated transition model supported this approach, emphasizing the importance of phased transitions with shared responsibility between pediatric and adult teams (23).

A 2019 assessment of our clinic found that 71% of patients (*N* = 75) transitioned successfully, defined as returning for more than one-follow-up appointment at the adult nephrology clinic after the initial transition clinic visit at Lurie Children's Hospital (16). After implementing enhanced communication protocols and structure support mechanisms, this rate improved to 92%. In a 2020 survey, 84% of patients reported feeling ready for transfer; however, only half felt fully confident navigating adult care independently, underscoring the importance of sustained support post-transfer (24).

### Structure tools and digital support improve engagement

To tailor the pace of transition to individual needs, we used the Transition Readiness Assessment Questionnaire (TRAQ) and had individual discussions about patients’ goals and self-management skills (25). These tools allow us to assess and build readiness systematically. In addition to in-clinic interventions, digital tools such as mobile health apps for medication reminders and symptom tracking represent a promising adjunct. While not formally implemented in our program, future integration of these technologies could enhance patient empowerment and treatment adherence.

### Multisystem monitoring and genetic counseling are essential components of adult care

As patients age, adult care must extend beyong nephrology management to include routine assessments for systemic complications such as hypothyroidism, pancreatic dysfunction, myopathy, and neurocognitive effects. Transition planning should also include education on the importance of reproductive health and the availability of genetic counseling for family planning. These aspects, while often underemphasized, are crucial in providing holistic, adult-centered care to this population.

### Psychological support and life planning must be prioritized

Transition-age youth with cystinosis often face mental health challenges, social isolation, and identify conflicts related to independence vs. medical dependence. While our clinic emphasizes open communication and regular check-ins, broader psychological interventions are also needed. Resources for structured mental health care, access to peer support groups and vocational/education counseling are ways to improve emotional well-being and long-term quality of life.

### Dedicated teams and consistent communication are the cornerstone of success

Our clinic's success has been rooted in a multidisciplinary team model, consistent communication among pediatric and adult providers, and proactive patient follow-up. We conduct monthly case reviews of all transitioning patients and maintain contact through personalized outreach–by phone, email, or text–when appointments are missed. This high-touch approach ensures patients are re-established after missed appointments and reinforces the patients’ sense of being supported during a vulnerable period.

### Future directions

Building on the challenges identified and lessons learned, future efforts should focus on developing standardized and evidence-based transition protocols tailored to rare, multisystemic diseases like nephropathic cystinosis. Expansion of integrated pediatric-adult care models will be essential to ensure continuity and trust. Digital health tools that support medication adherence and symptom tracking should be evaluated for efficacy in this population. Additionally, longitudinal studies are needed to assess the impact of structured transition programs on clinic outcomes, quality of life, and healthcare utilization.

## Conclusion

Structured transition can help patients with nephropathic cystinosis transfer successfully to adult providers. As cystinosis patients age, pediatric nephrologists will need to hand over the care of these patients to adult nephrologists who have little experience with this rare disease. Adult nephrologists, with the help of their pediatric colleagues and a multidisciplinary team, can effectively help cystinosis patients during the transition of care. A transition team can enhance the quality of care, improve patients’ sense of autonomy, and ultimately improve patient outcomes.
